# Evaluation of short read metagenomic assembly

**DOI:** 10.1186/1471-2164-12-S2-S8

**Published:** 2011-07-27

**Authors:** Anveshi Charuvaka, Huzefa Rangwala

**Affiliations:** 1 Computer Science Department, George Mason University, Fairfax, Virginia, USA

## Abstract

**Background:**

Metagenomic assembly is a challenging problem due to the presence of genetic material from multiple organisms. The problem becomes even more difficult when short reads produced by next generation sequencing technologies are used. Although whole genome assemblers are not designed to assemble metagenomic samples, they are being used for metagenomics due to the lack of assemblers capable of dealing with metagenomic samples. We present an evaluation of assembly of simulated short-read metagenomic samples using a state-of-art de Bruijn graph based assembler.

**Results:**

We assembled simulated metagenomic reads from datasets of various complexities using a state-of-art de Bruijn graph based parallel assembler. We have also studied the effect of k-mer size used in de Bruijn graph on metagenomic assembly and developed a clustering solution to pool the contigs obtained from different assembly runs, which allowed us to obtain longer contigs. We have also assessed the degree of chimericity of the assembled contigs using an entropy/impurity metric and compared the metagenomic assemblies to assemblies of isolated individual source genomes.

**Conclusions:**

Our results show that accuracy of the assembled contigs was better than expected for the metagenomic samples with a few dominant organisms and was especially poor in samples containing many closely related strains. Clustering contigs from different k-mer parameter of the de Bruijn graph allowed us to obtain longer contigs, however the clustering resulted in accumulation of erroneous contigs thus increasing the error rate in clustered contigs.

## Background

Advances in sequencing technologies have equipped researchers with the ability to sequence collective genomes of entire microbial communities, commonly referred to as metagenomics, in an inexpensive and high-throughput manner. Microbes are omnipresent within the human body and environments across the world. As such, characterizing and understanding their roles is crucial for improving human health and the environment. Metagenomics provides an unbiased view of the diversity and biological potential of microbial communities [1] and analysis of community samples from several different microbial environments has provided some key insights into the understandings of these microbial communities. Some of the important metagenomic endeavours have radically transformed our knowledge of microbial world. One of the pioneering studies, which sequenced samples from Sargasso Sea [2], revealed more than 1.2 million unknown genes and identified 148 new bacterial phylotypes. Another study of Sorcerer II Global Ocean Sampling project [3] has added many new protein families to the existing protein databases and a large scale metagenomic analysis of fecal samples [4] has identified and cataloged a common core of genes and gut bacteria.

One of the major challenges related to metagenomic processing is the assembly of short reads obtained from community samples. Due to the lack of specific assemblers to handle metagenomes, researchers continue to use assemblers originally developed for whole genome assembly.

We have evaluated the performance of a state-of-the-art Eulerian-path based sequence assembler on simulated metagenomic datasets using a read length of 36 base pairs (bp), as produced by the Solexa/Illumina sequencing technology. The datasets were meant to reflect the different complexities of real metagenomic samples [5]. They included, a low complexity dataset with one dominant organism, a high complexity dataset with no dominant organism and a medium complexity dataset having a few dominant organisms. We also created a dataset containing different strains of the same organism to measure the extent of co-assembly when reads from very similar organisms are used. Since the metagenomic read datasets are voluminous, we used a parallel sequence assembly algorithm (ABYSS [6]) which can be deployed easily on a commodity Linux cluster.

The assembled contigs were evaluated based on several quality measures for contig length and assembly accuracy. To improve the quality of the contigs, we clustered the results of different parameter runs of the assembler. We used efficient local alignment to quickly and accurately map the assembled contigs to the input source genomes. We also used a short read mapping algorithm to align the input reads to the assembled contigs to compute the homogeneity of the assembled contigs using entropy as a metric. Finally, we assessed the coverage of the source genomes by the produced contigs.

Short-read assembly of metagenomes performed better than our initial expectation in some aspects such as accuracy of the contigs and coverage of the source genomes. However, fragmentation of the contigs was more severe in metagenomic datasets than in the isolate assemblies. The assembly of a smaller dataset consisting of reads from 30 EColi strains showed that the contigs obtainable through co-assembly of related strains are considerably shorter than those generated using isolate assemblies. We also observed that by clustering results from assembly runs for different k-mer size values of de Bruijn graph, we were able to obtain a greater number of longer contigs (as optimal contigs are distributed across the k-mer space).

### Metagenomics overview

Traditionally, microbial genomics has relied primarily on pure cultures of microbes for sequencing. In recent years, researchers have developed a new approach known as metagenomics wherein the genetic material is obtained by directly sequencing the complex microbial communities without prior culturing. This presents an unbiased view of the diversity and biological potential of these communities [1].

The heterogeneous nature of the genetic material contained in metagenomic samples presents significant challenges for metagenomic assembly and analysis. Metagenomic samples have genomic content from many organisms which can not be easily separated. The genetic material of individual organisms in these samples is roughly proportional to the abundance of these organisms in the communities, which varies significantly. The dominant organisms are over-represented whereas the organism at low levels of abundance are not sequenced at sufficient depth. Also, the polymorphism between related members of the communities may lead to incorrect estimation of the repeat structures [7].

Phylogenetic classification of metagenomic reads is problem closely related to assembly. Ideally if the reads could be separated by their respective genomes the the problem of assembling them would be much more simple. However, segregation of reads in this manner is difficult to perform and several supervised and unsupervised approaches have been developed to address this problem. Some notable ones include Phymm and PhymmBL [8] which uses Interpolated Markov Models, MEGAN [9][10] which classifies sequences based on Lowest Common Ancestor from sequence similarity search using BLAST and PhyloPythia [11], a multiclass support vector based classifier using oligonucleotide frequencies. Unsupervised methods for classifying reads are sometimes also referred to as clustering or binning methods [12][13]. Unsupervised methods have an advantage over the supervised methods because the known sequences only represent a minority of the estimated microbial diversity [14]. The major drawback of classification and binning methods is that they are reliable only for relatively long sequences, of size greater than 1000 *bp*. In addition to binning and assembly, several other tools have been developed for gene prediction [15][16] and comparative analysis of metagenomic datasets [10][17]. We refer the reader to [18] for a review of computational challenges and available tools for metagenomics.

### Next Generation Sequencing and short read assembly

Sanger’s method [19] has been the dominant sequencing platform for several decades. In recent years, the emergence of the so called “Next Generation Sequencing” (NGS) [20] technologies has radically transformed DNA sequencing domain. These new technologies are amenable to parallel sequencing and yield a much higher throughput at significantly lower cost per base compared to Sanger’s method. The compromise with NGS is shorter read length which seems to be getting better gradually. NGS is particularly suited for metagenomic applications because it obviates the need for clonal culturing and the lower per base cost allows the genomes to be sequenced at much greater depth than feasible through Sanger-based methods. The conventional Overlap Layout Consensus (OLC) strategy has been one of the most successful paradigms for assembling long Sanger-based reads. However, in the recent years an alternative method inspired by the Sequencing by Hybridization and based on Eulerian tour of de Bruijn graphs has gained prominence. Some of the assemblers using this Eulerian-based approach include EULER [21], VELVET [22], ABYSS [6] and ALLPATHS [23].

### Related work

To study the extent of errors in metagenomic assemblies in comparison to single genome assembly, we performed a set of experiments on simulated datasets. Although, simulated datasets do not completely capture the characteristics of real metagenomes [24], simulation studies do provide some insight into the feasibility of assembly of short read metagenomic samples. In our current work, we have estimated the extent of problems associated with the assembly of short reads obtained from next generation sequencing Solexa platform for metagenomic samples. A similar study by Mavromatis et al. [5] produced three simulated metagenomic datasets representing microbial communities of different complexities using reads obtained from Sanger-based sequencing. They used these datasets for benchmarking various metagenomic processing methods. One of the focuses of their study was estimating the chimericity in assembling reads obtained from Sangers sequencing using Overlap-Layout-Consensus (OLC) based assemblers commonly used for whole genome assembly. Another simulation study by Wommack et al. [25] evaluated simulated NGS short reads from different metagenomic samples for taxonomic and functional annotation. As more and more metagenomic projects have started to tap into the potential of NGS, we felt the need for a similar simulation study to evaluate short read assemblers. The work of Pop [7] provides a good overview of OLC and Eulerian assembly paradigms and addresses some of the challenges associated with short read assembly. Since the next generation sequencing allows the samples to be sequenced at a greater depth, we used considerably larger datasets. Several researchers have studied the performance of NGS short reads and paired-end short reads for individual genome assembly [26-28]. Recently, Kingsford et al. [29] performed a theoretical analysis of Eulerian-path based approaches to survey the repeat structure of individual prokaryotic genomes.

## Results and Discussion

We have evaluated metagenomic assemblies based on the accuracy of the generated contigs using alignment-based similarity to the source genomes, contig length statistics, and the proportions of the source genomes recovered by the contigs. As the k-mer size of de Bruijn graph plays a crucial role in ABYSS’s assembly, we have also tried to assess the impact of k-mer size on metagenomic assemblies by comparing the contigs produced from different runs of the k-mer parameter. We also provide comparisons of metagenomics assemblies to the isolate assemblies of the constituent genomes.

### Impact of de Bruijn graph k-mer size on contig length

**Figure 1:**
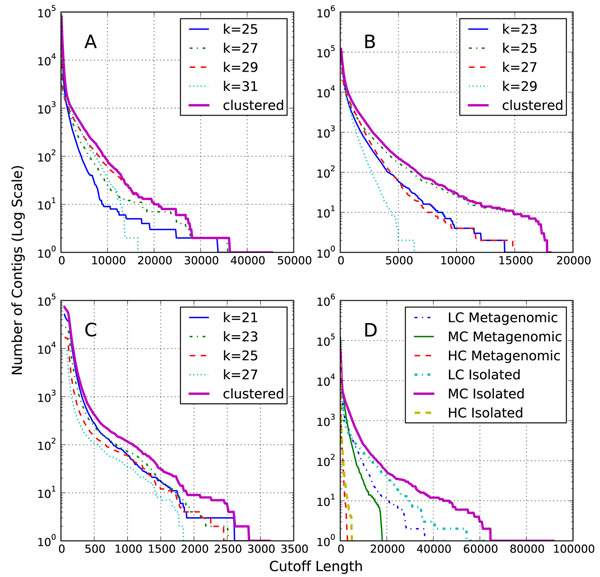
**Contig length distribution** The total number of contigs (in log scale) shorter than a given cutoff length, for each of the datasets. (A) simLC. (B) simMC. (C) simHC. (D) Comparison of length distribution of the clustered contigs for metagenomic and isolate assemblies.

**Figure 2:**
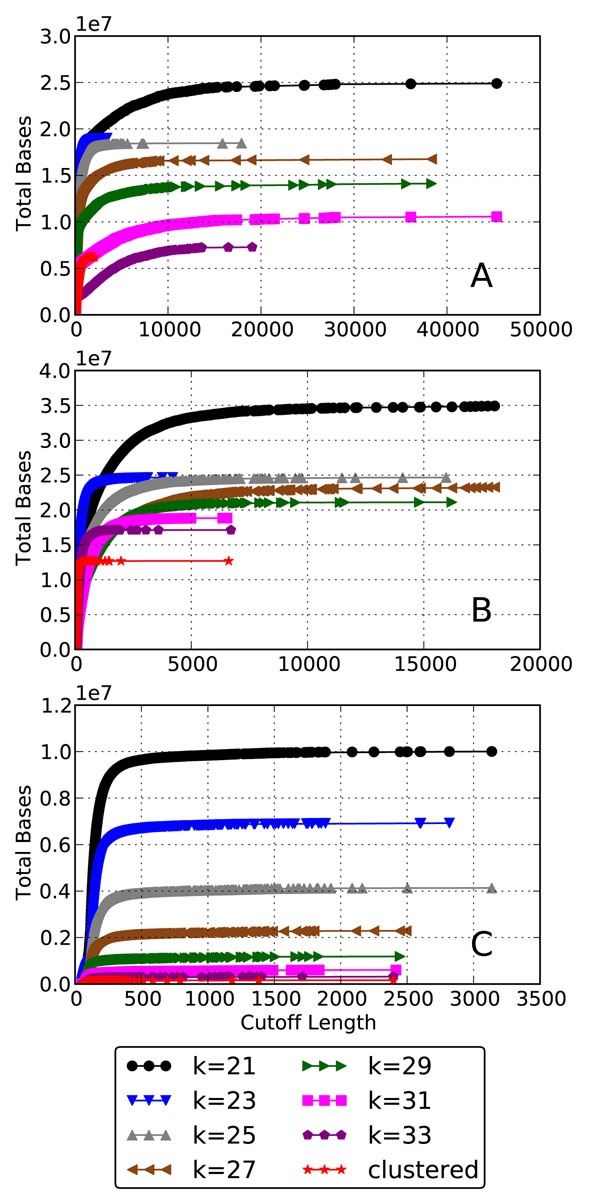
**Total bases recovered at different contig length cutoffs** The total number of bases contained in all the contigs shorter than a certain cutoff length (A) simLC. (B) simMC. (C) simHC.

Figure 1 shows a comparison of the contig lengths to k-mer size across the simLC, simMC and simHC datasets. The average lengths of the contigs decrease with an increase in the complexity of the dataset. From Figure 1 it can be seen that the optimal value of *k* to obtain longer contigs changes from 29 for simLC (a single dominant genome at a very high coverage) to 21 and 23 for simHC, which does not have any distinctly dominant genomes. As seen from Figure 1(B), the simMC dataset *k* = 25 seems to produce the longer contigs. The clustered results effectively pool the contigs produced from different runs of assembly by varying the k-mer parameter values across the different datasets. Figure 2 shows the distribution of number of bases recovered by the contigs for different cutoff lengths of the contigs and different runs of the assembly. It can be seen from the figure that in general the shorter contigs account for majority of the bases recovered. In all the cases, the number of bases recovered increased with the use of smaller values of k-mer parameter as a greater fraction of the low coverage genomes were being assembled. Table 1 summarizes these statistics regarding the assembled contigs and also provides *N*50 (weighted median) values.

**Table 1:** Contig Alignment Statistics.

Dataset	K	Total Contigs	Accurate Contigs	% Accurate Contigs	N50	Total Bases	Bases in Accurate Contigs	% Bases in Accurate Contigs
	**C**	81451	74790	91.82	466	24892639	24163037	97.07
	**C-21**	67448	66144	98.07	447	21509228	21300001	99.03
	21	125340	119834	95.61	191	19014129	18475501	97.17
	23	74630	73850	98.95	325	18475496	18350279	99.32
simLC-36m	25	69028	68731	99.57	279	16751244	16697244	99.68
	27	68245	68010	99.66	206	14123037	14087137	99.75
	29	52885	52765	99.77	302	10562147	10545749	99.84
	31	26382	26339	99.84	2363	7276731	7272359	99.94
	33	27332	27306	99.9	340	6214325	6211238	99.95

	**C**	119667	112827	94.28	493	34920211	34050685	97.51
	**C-21**	100852	98658	97.82	566	31992027	31595445	98.76
	21	183383	178586	97.38	161	24663986	24173013	98.01
	23	106981	106133	99.21	324	24661676	24510881	99.39
simMC-36m	25	88466	88122	99.61	419	23280192	23216974	99.73
	27	78074	77825	99.68	569	21119299	21075047	99.79
	29	75800	75571	99.7	400	18839097	18777496	99.67
	31	114336	113948	99.66	168	17128489	17050171	99.54
	33	156709	156272	99.72	78	12688930	12646726	99.67

	**C**	73480	55508	75.54	138	10007373	7649152	76.44
	**C-21**	39196	35369	90.24	131	5366108	4823423	89.89
	21	51371	36693	71.43	142	6923506	5037993	72.77
	23	28614	25707	89.84	137	4132863	3703686	89.62
simHC-36m	25	17418	16557	95.06	122	2289332	2179104	95.19
	27	9822	9524	96.97	109	1184664	1149541	97.04
	29	5309	5211	98.15	102	603680	593152	98.26
	31	3047	3005	98.62	93	315736	311501	98.66
	33	1895	1885	99.47	77	162625	161704	99.43

	**C**	25742	25359	98.51	1223	9985001	9913743	99.29
	21	24883	24709	99.3	544	6660066	6627437	99.51
	23	20550	20459	99.56	847	6560491	6545844	99.78
EcoliStrains-10m	25	19570	19506	99.67	933	6370414	6356780	99.79
	27	17474	17422	99.7	1195	5995915	5986494	99.84
	29	17338	17278	99.65	925	5560578	5550393	99.82
	31	25468	25436	99.87	317	5237879	5233758	99.92

simLC-36m, simMC-36m, simHC-36m are the results for the low, medium and high complexity datasets with 36 million reads, respectively. EcoliStrains-10m are the results for the co-assembly strain dataset with 10 million reads. **C** shows the clustering results after pooling contigs obtained from running ABYSS with *k* ranging from 21 to 33. **C-21** shows the clustering results after excluding the contigs obtained by running ABYSS for *k* = 21.

### Comparison of metagenomic assembly to isolate assembly

As a benchmark for our metagenomic assemblies we separated the reads by their source sequence and performed isolate assemblies. We assembled the reads from each individual sequence separately and combined the final contigs from all the source sequences. We performed the isolate assemblies with different values of *k* and pooled the results using the clustering algorithm. Figure 1 (D) compares the length distribution of clustered results form metagenomic and isolate assemblies. The simHC dataset produced shorter contigs in both isolate as well as metagenomic assemblies. Amongst the simLC and simHC datasets, the performance of simLC was closer to the isolate assemblies, whereas, the simMC metagenomic assembly was far poorer in comparison to its isolated assembly.

### Contig alignment accuracy

Even assemblies of isolate genomes are not completely error free. In the case of metagenomes, the presence of multiple genomes at different coverage depths causes additional problem and the contigs are expected to have more mis-assemblies compared to the contigs from isolate genome assemblies. To assess the accuracy of the assembly we aligned the contigs back to the source genomes. Table 1 reports the results for the different datasets. A threshold accuracy of 95% was used for considering a contig accurate. Details of alignment methods and accuracy calculations are provided in the methods section. The assembly accuracy decreased as the k-mer size was decreased and was worst for all datasets at *k*=21. Further, the accuracy of the clustered contigs was lowest, due to the accumulation of errors from all the contig sets. This is due to our clustering approach, which tries to retain all the unique sequences. An alternative clustering strategy could be designed that takes the consensus of contigs obtained from different runs. This strategy would improve the accuracy of the results while reducing the total number of bases recovered.

### Contig homogeneity

In an ideal case of metagenomic assembly, all the reads forming a contig would come from the same source sequence. In metagenomes the reads from different sources may be co-assembled, resulting in chimeric assemblies. Therefore, to estimate the degree of chimericity, we evaluated the homogeneity of contigs using their read composition. Essentially, greater the number of sources from which a contig is assembled, the higher its entropy will be. The methods section describes the alignment of reads to the assembled contigs and entropy calculations.

**Figure 3:**
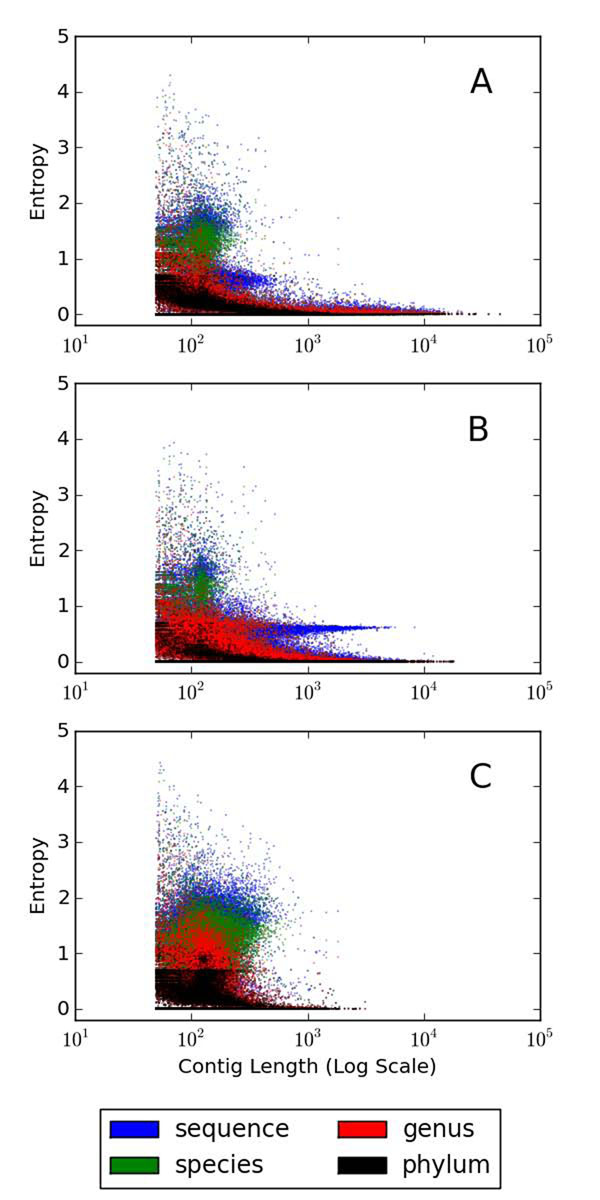
**Contig entropy at different taxonomic levels** The entropy of contigs versus the contig length (in log scale) for the datasets (A) simLC. (B) simMC. (C) simHC.

Figure 3 shows a plot of contig entropy versus length of the contigs across the datasets of different complexities. The entropy metric is computed at four levels: (i) sequence, (ii) species, (iii) genus and (iv) phylum, derived from the NCBI taxonomy tree. The simHC dataset produces a large number of smaller inhomogeneous contigs due to insufficient coverage of the source sequences. The proportion of inhomogeneous contigs is comparatively lower in the MC and significantly lower in LC datasets. The contigs were more homogeneous at higher phylogenetic levels. Because the genomes which are phylogenetically close together share significant sequence similarity, there is a greater chance of assembling reads belonging to related sequences into the same contig.

### Coverage of the source sequences

**Figure 4:**
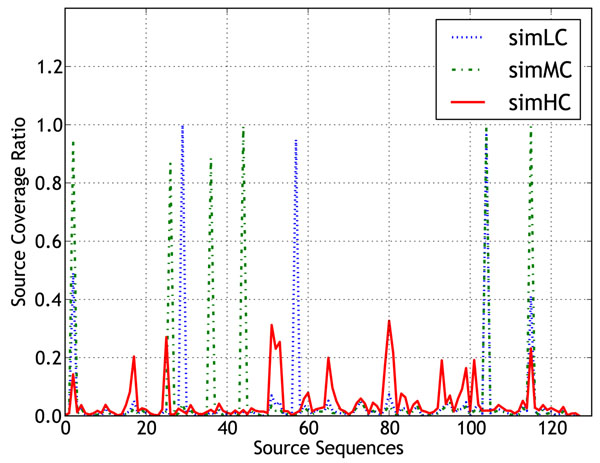
Coverage of source sequences from the clustered contigs.

Figure 4 shows a plot of the source coverage ratio for the clustered contig sets of simLC, simMC, and simHC datasets. The positions of the source sequences in Figure 4 correspond to those in the read coverage plot discussed in methods section. Due to space constraints, we did not include the plots showing the coverage values for different k-mer sizes, but we summarize the results here. In almost all the assemblies a high proportion of source genomes sequenced at higher depth was recovered by the contigs. As the value of k-mer size was decreased, more of the genomes sequenced at lower depth were recovered. However, as evident from the contig length distribution plot, Figure 1, some values of k-mer size tend to be suboptimal length-wise, depending on the complexity of the datasets. Therefore, clustering of the contigs resolves this issue, as the clustered results retain the longer contigs and also the unique contigs representing the low read coverage genomes.

### Escherichia strains co-assembly

**Figure 5:**
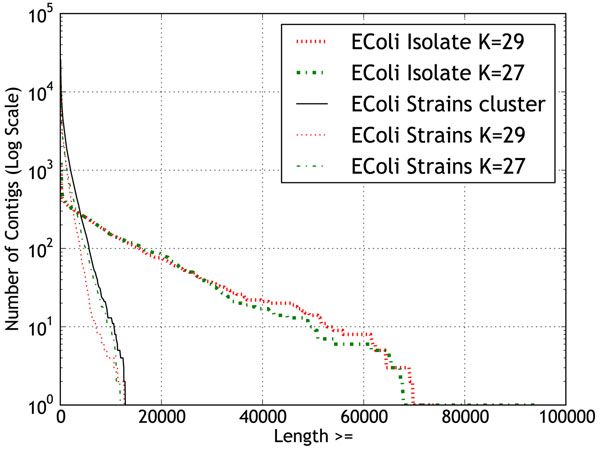
**Contig length distribution of EcoliStrains dataset and isolate assembly.** The total number of contigs (in log scale) shorter than a given cutoff length

**Figure 6:**
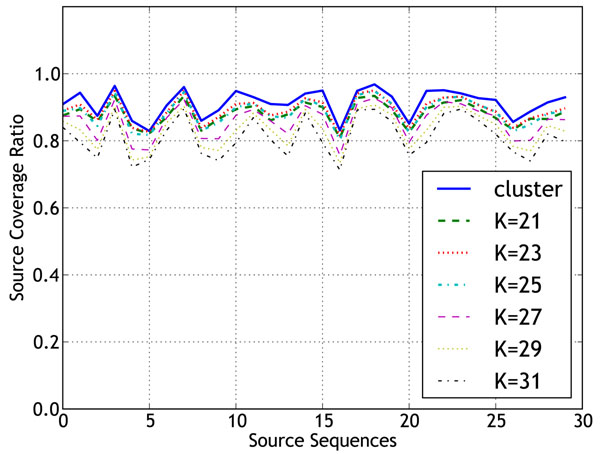
Coverage of source sequences for EColi Strains

Since the collection of DNA sequences for metagenomic experiments does not involve cloning, the reads could come from strains which are highly similar, with very little sequence variation. In this case, even though the effective read coverage of the species is high, due to minor differences in the sequences of the strains, the quality of assembly might not be as good as an isolate genome assembly. To evaluate the performance of co-assembly of reads from related strains, we created the EColiStrains dataset consisting of 10 million reads from 30 strains of Escherichia Coli. For comparing the assembly performance, we created another dataset with the same number of reads from a single strain, E.Coli strain 536 (represents the isolate assembly), and assembled it using the different k-mer size values used for assembly of the strains dataset. For isolate assembly, *k*=27 and 29 produced the longest contigs. Figure 5 shows a comparison of isolated and metagenomic strains datasets for *k*=27 and 29. The contigs in metagenomic assembly are considerably shorter than the isolate assembly, suggesting a severe degradation in assembly quality resulting from the presence of multiple strains. Figure 6 shows the source coverage ratio of the constituent strains for different k-mer size values. A relatively high percentage of the source sequences was recovered by the contigs. Table 1 provides some additional assembly statistics for EColiStrains dataset. The EColiStrains dataset exhibited some of the same general trends as the simLC, simMC, and simHC datasets. But, the variations in the total number of bases and contig accuracies were less pronounced.

### Paired ended assembly

To evaluate the improvement in assembly quality with mate-pairs information we generated and assembled datasets similar to simHC, simLC and simMC with paired-ended reads of insert length 2000 bases. However we observed that only a small fraction (less than 2 %) of the contigs being produced were using mate pairs information. In addition, most of these contigs were assembled with gaps. For our analysis we broke those contigs at the gaps and treated them as separate contigs. Therefore, because to these two factors, we did not observe a significant improvement in the assembly quality of paired ended reads in metagenomic samples.

## Conclusions

In this paper we have presented the results of assembly and analysis of some simulated metagenomic datasets. Short-read assembly of metagenomes performed better than our initial expectations in some aspects such as accuracy of the contigs and coverage of the source genomes. Although a large fraction of the contigs were assembled accurately, fragmentation of the contigs was more severe in metagenomic datasets when compared to the isolate assemblies. Further, assembling the high complexity dataset was more difficult in comparison to the the low complexity dataset as well as the medium complexity datasets. We also observed that ABYSS was able to utilize the mate pairs information to assemble only a small fraction of the contig with gaps. Therefore, using mate-pairs did not improve the results significantly in our case. The assembly of a smaller dataset consisting of reads from 30 EColi strains showed that the contigs obtainable through co-assembly of related strains are considerably shorter than those generated using isolate assemblies.

We also observed that by simple clustering of the results from various assembly runs (with different k-mer size values of de Bruijn graph) we were able to obtain a greater number of longer contigs, as optimal contigs are distributed across the k-mer space. However, due to our simple approach towards clustering which retains all unique contigs, most mis-assembled contigs made their way into the clustered results, increasing their error rate. Further improvements in clustering technique may be required to improve the quality of the clustered results.

## Methods

### Datasets

We created our simulated datasets using Metasim [30]. It is a sequencing simulation tool for generating synthetic metagenomic datasets using a collection of complete genomic sequences. Metasim provides options for controlling various simulation parameters such as sequencing platform, read length, sequencing depth of individual sequences, error rate, and error distribution. We generated reads of length 36 bp using the default empirical error model of Metasim, which simulates the reads produced by Solexa sequencing technology. The bacterial sequences for generating the reads were taken from the completely assembled bacterial genomes from NCBI [31] genomes database.

Metagenomes vary considerably in their compositions depending on the environment from which the reads were sampled. Therefore, to assess the assembly quality as a function of metagenome’s complexity, we constructed three datasets using the profiles described in [5]. These datasets, **simLC** (low complexity), **simMC** (medium complexity), and **simHC** (high complexity) simulate the composition of real metagenomic datasets. In the low complexity simLC dataset, a sizable portion of the reads belong to a single dominant organism. The high complexity dataset simHC has no distinctly dominant organism and all organisms are present in approximately equal concentrations. The simMC dataset has more than one dominant organism, but their concentrations in the samples are considerably lower than the concentration of the dominant sequence in simLC. We also produced datasets similar to each of the three datasets described above with paired-end reads with an insert size of 2 kb, to evaluate the performance of paired-ended assembly.

**Figure 7:**
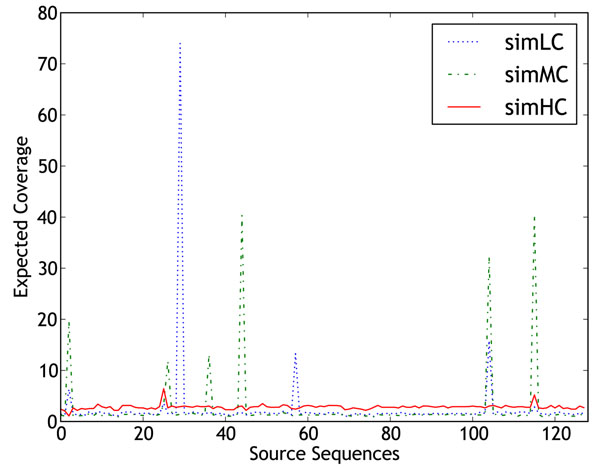
**Read coverage distribution** Distribution of the sampling depth of each genome in the datasets simLC, simMC and simHC.

Figure 7 shows a plot of sequencing depth of individual sequences for these three datasets. Each of these datasets contain 36 million reads taken from 128 sequences belonging to 113 organisms. The combined sequence data contained in each dataset was approximately 1300 Mb, which is equivalent to the amount of data produced by a single run of a Solexa sequencer. Some of the sequences used in [5] are still in the draft assembly stage, and therefore, to retain the same levels of complexity in our datasets, we replaced the missing genomes with the phylogenetically closest completely assembled sequences from NCBI. Additional information related to the datasets has been made available at the supplementary website [32]. We constructed a fourth dataset, **EcoliStrains** consisting of 10 million reads sampled uniformly from 30 different strains of *Escherichia coli*. The coverage of each strain was approximately 2.3x. This dataset was constructed to study the extent of co-assembly when reads from very similar organisms are assembled together.

### Assembly

Due to the high computational requirements for the assembly of our metagenomic datasets, we used ABYSS [6] assembler (version - 1.0.8) which can perform parallel assembly using a cluster of commodity computers. We assembled all of our datasets using ABYSS, with read length of 36 bp and varied the k-mer size parameter of ABYSS’s distributed de Bruijn Graph between 21 and 33 (in increments of two) to obtain different assemblies. In the presence of sequencing errors the optimal k-mer size for Eulerian path based assemblers is determined by the coverage of source sequences. For high coverage, values close to read length produce longer contigs. Similarly, if the percentage of sequencing errors is high, optimal results are obtained by decreasing the k-mer size. All the assembly jobs were run using 32 cluster nodes. We filtered out contigs shorter than 50 bp from the final assemblies.

We also performed paired-ended assemblies using ABYSS on datasets generated with mate-pairs information. ABYSS performs paired-ended assembly in two stages, first it assembles the reads without using the mate-pair information and in the second stage it utilizes the paired reads information to merge the contigs. We set the minimum number of pairs required to merge the contigs at 10.

Due to the presence of sequencing errors and repeat regions in the source genome, assemblies are usually not completely error free, even in the context of a single genome. We assembled the reads from the individual source genomes by separating them first and combined all the produced contigs. In this study, the assembly performed in this manner is referred to as isolate assembly and provides us a comparative baseline to the metagenomic assembly.

### Clustering assembled contigs

We observed that the contigs of optimal length were distributed across the k-mer space. Therefore, we pooled the assembled contigs from different contig sets (obtained using different k-mer values) and clustered them to remove duplicate or suboptimal contigs which were contained in another longer contig. We clustered the contigs using Cd-hit [33], which uses a greedy incremental algorithm. The first cluster is formed using the longest sequence as the cluster representative, and the remaining sequences are compared to it in decreasing order of their lengths. If a sequence matches to one of the cluster representatives with sufficient accuracy, then it is placed in that cluster. Otherwise, a new cluster is formed with the unmatched sequence as the cluster representative. Instead of performing the actual alignment, Cd-hit uses a short word filtering algorithm to compute sequence similarity, therefore, it achieves significant speed-up compared to alignment based clustering tools. We clustered our assemblies using a similarity threshold of 95% and a word size of 8 bases (recommended for clustering with high similarity).

### Alignment of contigs to reference genomes

To estimate the assembly accuracy we aligned the contigs to the source genomes. Accurate contigs are expected to match at least one source sequence with high accuracy. Therefore, to speed up the alignment process we used NUCMER pipeline of MUMMER [34]. NUCMER uses a suffix tree based string matching algorithm to search for exact matches and extends these matches using a dynamic programming based alignment and is considerably faster than BLAST. We set the parameters for exact match size to 15 and minimum cluster size to 30 and collected all possible matches, so that the sensitivity is not significantly affected. We calculated the contig accuracy by normalizing the accuracy of the local alignment (*localAcc*) produced by NUCMER using the ratio of length of the alignment (*alignLen*) to the length of the contig (*contigLen*), as given by (1).(1)

Some contigs, especially the shorter ones, produced multiple alignments, either to the same or to a different genome. Therefore, we used the best accuracy among all the alignments as the contig’s assembly accuracy. For contig coverage calculations (discussed later on) we consider only the contigs that were assembled with a threshold accuracy of at least 95%. We would like to note that this threshold is rather arbitrary and in many cases, the acceptable accuracy threshold would be dependent on particular application. Our choice was motivated by a similar threshold used by the authors of ABYSS [6] for evaluating the assembly accuracy for isolate genomes.

### Contig homogeneity calculations

We estimated the homogeneity of contigs by observing the source genome of reads used to assemble a contig. This was done by performing a read-to-contig alignment using a fast short read aligner. We used BWA [35], which performs a backward search with burrows wheeler transform and efficiently aligns short reads against reference sequences. In our case, the references consisted of the set of contigs. Each read was assigned to the contig to which BWA reported the best match.

Using the counts of reads from each source sequence mapped to a given contig, we calculated the entropy of the contigs as shown in (2).(2)

where *p_i_* is the fraction of total reads coming from source genome *i*. At different phylogenetic levels, organisms generally display a greater sequence similarity within their group when compared with the organisms belonging to a different group. Due to this sequence similarity, the assemblers are more likely to make the mistake of mis-assembling reads belonging to the same phylogenetic class. We also compute the entropy at two higher phylogenetic levels, genus and phylum, in addition to the entropy at sequence and strain level, to see if there is a significant decrease in entropy at higher phylogenetic levels.

The need for a short-read aligner arises because, for Eulerian path based assemblers, it is difficult to determine the actual read composition of the contigs. The input reads are not used directly but are broken down into smaller k-mers and the original read information is lost.

### Source coverage ratio

For different assemblies generated by varying the values of assembly parameter *k*, we calculated the extent to which the source sequences are recovered by the contigs. This is determined by aligning the contigs to the source sequences. We considered only the accurate alignments of the contigs, i.e. the alignments which accurately cover at least 95% of the contig. For each such alignment, we marked all the positions of the source genomes which were part of the alignments. The collection of all such positions of the source genome covered by the contigs, represents the contig coverage of the genome. The contig coverage described here is different from the read coverage which is approximate coverage of the genome from the sampled reads and represents the sequencing depth of the source sequences in the datasets. The contig coverage represents the fraction of the source sequence recovered by the contigs and can be at most 1. We refer to this contig coverage as the *source coverage* ratio.

In cases where contigs had multiple accurate alignments, possibly due to repeat regions or shared sequences between genomes, we counted each contig’s contribution for all the alignments. Therefore, our contig mapping to source genomes is not unique, and our source coverage ratio calculation may have over-counted a little. As it is not possible to prefer one particular alignment over another, we believe this is a better option than randomly choosing a particular alignment of the contig.

## Authors contributions

AC performed the experiments and wrote all the programs needed for this study. Both, AC and HR conceptualized this study and setup the different experiments. All authors wrote the manuscript, read the manuscript, and approved for final submission.

## Competing interests

The authors declare that they have no competing interests.
